# Hemoglobin Levels in Children Treated for Cystic Fibrosis with CFTR Modulators: A Single Center Retrospective Study

**DOI:** 10.3390/jcm14144856

**Published:** 2025-07-09

**Authors:** Antonella Tosco, Raffaele Cerchione, Monica Gelzo, Chiara Cimbalo, Alice Castaldo, Rosamaria Terracciano, Valeria Raia, Angela Sepe

**Affiliations:** 1 Paediatric Unit, Cystic Fibrosis Regional Reference Center, Department of Maternal and Child Health, University Hospital Federico II, 80131 Naples, Italy; antonellatosco@gmail.com (A.T.); ornellasepe@hotmail.com (A.S.); 2 Paediatric Unit, Cystic Fibrosis Regional Reference Center, Department of Translational Medical Sciences, University of Naples Federico II, 80131 Naples, Italy; raffaele.cerchione@outlook.it (R.C.); rosamariaterracciano@libero.it (R.T.); raia@unina.it (V.R.); 3 Centro di Ingegneria Genetica e Biotecnologie Avanzate F. Salvatore (CEINGE) Advanced Biotechnology Franco Salvatore, 80145 Naples, Italy; monica.gelzo@unina.it; 4 Department of Molecular Medicine and Medical Biotechnology, University of Naples Federico II, 80131 Naples, Italy; 5 Pediatric Pneumology and Sub Intensive Care and Rehabilitation Unit, Santobono-Pausillipon Hospital, 80129 Naples, Italy; ali.castaldo@hotmail.com; 6 Department of Public Health, University of Naples Federico II, 80131 Naples, Italy

**Keywords:** elezacaftor/texacaftor/ivacaftor, lumacaftor/ivacaftor, hemolysis

## Abstract

**Background:** An increase in hemoglobin (Hb) has been reported in subjects with CF treated with the CFTR modulator Ivacaftor and with the combination Lumacaftor/Ivacaftor (LI), while the literature about the impact of Elexacaftor/Tezacaftor/Ivacaftor (ETI) on Hb levels in the pediatric population is lacking. **Materials and Methods:** We retrospectively evaluated Hb levels in 35 subjects with CF (18 males, median age: 8 years; interquartile range (IQR): 6–13 years) treated with LI and 60 (24 males, median age: 10 years; IQR: 6–14 years) treated with ETI. For each subject we considered the values of Hb, serum potassium, total bilirubin (TB), and conjugated bilirubin (CB) at baseline, after 3 days, and 1, 3, 6, 9, and 12 months from the start of treatment. **Results:** In subjects with CF treated with LI, we observed a significant increase in Hb values 3 days after the introduction of the drug, which remained constant throughout the year of treatment. In subjects treated with ETI, a significant decrease in Hb was observed 3 days after the first dose up to 1 month. At 6 months, Hb returned to pre-treatment values remaining stable for up to 12 months. At 3 days of treatment, we also observed a significant increase in serum potassium, which returned to normal at one month, while both TB and CB values significantly increased at 3 days of treatment and remained significantly higher for the whole one-year period of ETI therapy. **Conclusions:** We confirmed an increase in Hb values over time in subjects treated with LI. While the Hb response in those treated with ETI showed a transient reduction that lasted for one month, this may have depended on hemolysis, and returned to pre-treatment levels. Further studies will clarify the mechanisms that govern changes in Hb in subjects with CF treated with ETI.

## 1. Introduction

Cystic fibrosis (CF) is a progressive inherited disease with an autosomal recessive pattern and affects more than 100,000 people worldwide. The disease is caused by over 2000 known mutations in the *Cystic Fibrosis Transmembrane Regulator* (*CFTR*) gene, which encodes for CFTR protein [1]. In physiological conditions, CFTRs work as a chloride/bicarbonate channel expressed at the apical plasma membrane of several epithelial tissues, where it regulates the composition and quantity of epithelial secretions. CFTR mutations impair CFTR protein expression and function, leading to an accumulation of thick mucus, recurrent infections, and systemic chronic inflammation. Pulmonary and extrapulmonary clinical features may occur in subjects with CF.

The role of CFTRs in erythrocytes is still unknown. During their passage through capillaries, it has been observed that CFTRs facilitate erythrocytes deformation, which triggers ATP release. Erythrocytes from subjects with CF who carry the F508del mutation show a reduced distribution of CFTRs on the plasma membrane [2]. It is well known that iron deficiency is a common extrapulmonary manifestation that could be involved in the development of anemia in CF, especially in adolescents and adults with advanced diseases [3,4]. Chronic inflammation could play a crucial role in the multifactorial aetiology of anemia in CF, leading to anemia typical of chronic disease, where inflammatory cytokines disrupt iron metabolism and suppress red blood cell production [5].

In addition, malabsorption of key nutrients, like iron, vitamin B12, folate, and fat-soluble vitamins (such as vitamin E), due to pancreatic insufficiency can contribute significantly to anemia in CF patients. Gastrointestinal blood loss and poor dietary intake may also exacerbate the problem. Recurrent infections and the body’s heightened immune response further stress the bone marrow’s ability to produce healthy red blood cells.

Anemia in CF has been associated with more severe lung disease and reduced pulmonary function, and particularly with lower FEV_1_ values. It may also reflect the overall burden of systemic inflammation and infection. Fatigue, reduced exercise tolerance, and diminished quality of life are common symptoms, although mild anemia can go unnoticed without routine blood work [6].

In the last decade, the care of subjects with CF has improved significantly thanks to CFTR modulators, which are innovative therapies aimed at correcting or even enhancing the expression, function, and stability of defective CFTRs [1]. These drugs improve lung function and nutritional status and reduce sweat chloride values, resulting in a better clinical status and quality of life. The potentiating modulator Ivacaftor (IVA) can currently be prescribed to children from 4 months of age who carry at least one gating mutation, and has a good safety and efficacy profile. The combination Lumacaftor/Ivacaftor (LI) is currently available for children aged 1 year and older who are homozygous for the F508del mutation. Among CFTR modulators, the combination Elexacaftor/Tezacaftor/Ivacaftor (ETI) is currently available for children with CF who carry at least one F508del variant, and leads to significant improvement in lung function tests and quality of life indicators from 6 years of age. This new modulator has an overall safety profile, but its long-term response is still unknown. Post-marketing reports and ongoing registry studies are needed to clarify these concerns. The numerous effects of CFTR-modulating drug therapy on extra-pulmonary manifestations of CF and comorbidities are currently being evaluated [7]. As survival improves for CF, attention to systemic complications, including anemia, becomes increasingly important. Early recognition and appropriate management of anemia can contribute to better respiratory outcomes that improve energy levels and overall quality of life.

Recently, in a study based on CF Foundation Patient Registry data of a population of adults and children with CF treated with IVA and LI, Gifford et al. demonstrated an increased hemoglobin (Hb) concentration during treatment [8,9]. In particular, using multivariate models, the authors demonstrated that IVA positively affected Hb in males, but not in females, while LI positively affected Hb in both sexes. Recent studies have shown how ETI, in a population of adult subjects, was able to significantly increase iron and ferritin status, Hb levels, and MCV values 2 years after the start of treatment [10,11]. To date, studies on early changes in Hb levels and on the pediatric population treated with ETI are lacking.

Therefore, we carried out a retrospective observational study to confirm the increase in Hb levels in children treated with LI and to observe how Hb levels change in children treated with ETI.

## 2. Materials and Methods

This was a single-centered retrospective study. All children and adolescents in regular follow-up at the Regional Cystic Fibrosis Center of Naples treated with LI or ETI for whom at least one year of observation was available were enrolled. Subjects without cystic fibrosis and with normal biochemical and hematological profiles were selected as healthy subjects (HCs). For each CF patient, a respective HC was selected by matching for sex and age (plus or minus one year). Informed consent was obtained from all patients (or from their legal guardians) for the use of anonymous clinical data for research purposes. This study was approved by the Ethical Committee of the CF Regional Centre of Campania (Ethics Committee number 77/2021).

Children and adolescents in regular follow-up at our center in order to start CFTR-modulators therapy were admitted to the hospital. For each child, routine laboratory checks were performed before starting therapy and 3 days after the beginning of treatment to evaluate any changes in biochemical parameters. Laboratory checks were repeated after 1 month and subsequently every 3 months. For the purpose of our work, for each group of subjects treated with modulators, Hb levels were evaluated before starting therapy (T0), in the early phase of the treatment (i.e., after 3 days and after 1 month), and at 3, 6, 9, and 12 months. Additionally, some other biochemical parameters, such as serum potassium, total bilirubin (TB), and conjugated bilirubin (CB), were evaluated at each time-point as indirect markers of hemolysis.

The Shapiro–Wilk test was applied to evaluate the normality of data distributions. Continuous non-parametric data were reported as median (interquartile range, IQR). Each comparison between the HC group and a single time point of LI (or ETI) treatment was evaluated by the Mann–Whitney U test. Paired comparisons between the different time points of LI (or ETI) treatment were performed using the Friedman test; *p*-values were adjusted for multiple testing using the Bonferroni correction. Statistical analyses were performed using SPSS (version 29); *p* values < 0.01 were considered significant.

## 3. Results

At the Regional Reference Center for the treatment of CF of Naples, 35 subjects with CF (18 males, median age: 8 years; interquartile range (IQR): 6–13 years) treated with LI and 60 (24 males, median age: 10 years; IQR: 6–14 years) treated with ETI were enrolled. We collected data for Hb at the baseline, in the early phase of the treatment (i.e., at 3 days, 1 month, and 3 months, Table 1A), at 6 and 9 months, and at one year after the beginning of therapy (Table 1B). In all subjects, Hb values were lower at the baseline compared to those of HCs (Table 1A). At 3 days of treatment, values of Hb significantly increased compared to the baseline and to HC values. After 1 month of treatment, Hb values were significantly lower compared to values at 3 days. After 3 and 6 months of treatment (Table 1A,B), values of Hb were significantly higher compared to the baseline and remained unchanged at 9 months and one year. Data about serum K, TB, and CB showed no significant difference through the follow-up period.

Sixty children and adolescents with CF were treated with ETI. We collected data regarding Hb, serum K, TB, and CB at the baseline, in the early phase of the treatment (i.e., at 3 days, 1 month, and 3 months) (Table 2A), at 6 and 9 months, and at one year (Table 2B). All values at the baseline were not significantly different in 60 CF subjects as compared to HCs (Table 2A). After 3 days of treatment, values of Hb were significantly lower as compared to those of HCs and to values at the baseline. Serum K values were significantly higher compared to those of HCs; TB and CB values were significantly higher compared to HC values. At one month, Hb was still significantly lower compared to baseline values, while serum K was significantly reduced compared to values at 3 days. Both TB and CB were significantly further increased. At 3 months, as during the long-term follow-up period, values of Hb and K did not change further, while values of TB and CB remained significantly higher compared to both HC and baseline values.

## 4. Discussion and Conclusions

According to the literature, our results highlight that in children and adolescents with CF, Hb values were significantly lower as compared to sex- and age-matched healthy control subjects and significantly increased during treatment with LI [2]. In our population, the increase was already evident after 3 days of therapy and remained rather constant over time, except for one month of therapy reaching values similar to the baseline. It is known that CFTR protein is expressed on erythrocytes’ plasma membrane, regulating the development, function, and/or survival of erythrocytes [9]. In CF subjects, anemia is the result of multifactorial events due to CFTR involvement in hematopoiesis, iron deficiency, and chronic inflammation. Managing anemia in CF requires a nuanced approach. Iron supplementation can be beneficial, but must be administered with caution, as excess iron may promote bacterial growth and worsen infections, particularly with pathogens like *Pseudomonas aeruginosa*. For this reason, decisions regarding treatment should be individualized based on the underlying cause of anemia and the patient’s clinical status [12].

Regular monitoring of hemoglobin levels, iron parameters (such as ferritin and transferrin saturation), and inflammatory markers is essential [13]. The improvement of constitutive inflammatory status and iron metabolism in CF subjects treated with modulators such as LI could lead to increased Hb levels.

As mentioned, to date the impact of ETI has only been evaluated on an adult population after 2 years of treatment, with the detection of increases in iron status and Hb and MCV values [10,11]. In contrast, changes in Hb values in the pediatric population treated with ETI are not yet clarified. In our population of children and adolescents, ETI significantly reduced Hb values after 72 h of therapy and for at least one month, even if the reduction was mild (not more than 20% of baseline values, and in no cases was the reduction so severe as to require therapy interruption). These values returned to those of pre-therapy after 6 months of treatment and remained stable over time. Even in a longer observation period for about half of these subjects, there did not seem to be significant increases. The mild early reduction in Hb, not previously described in any study of CF patients treated with ETI, was associated with a significant increase in the value of serum K after 3 days of treatment, suggesting a transient hemolytic effect. In our patients treated with ETI, we also observed a significant increase in both serum TB and CB at 3 days that persisted for the therapy’s duration, while Hb levels normalized, suggesting that the two alterations were not related. An increase in serum bilirubin in patients treated with ETI was observed by several studies and was attributed to Gilbert syndrome and the inhibition of bilirubin transporters, specifically organic anion transporting polypeptide B1 (OATP1B1) and organic anion transporting polypeptide B3 (OATP1B3) [14]. It may also be due to liver damage caused by liver inflammation triggered by cholesterol accumulation [15]. In children on ETI treatment, it is possible that mildly decreased Hb levels may have been due to Elexacaftor or Tezacaftor, as this event did not occur in children treated with LI. We are aware that our study was a monocentric study with a small sample size. Nevertheless, to our knowledge this is the first study to strictly evaluate changes in Hb values in a population of children and adolescents with CF treated with ETI. According to the technical sheet, biochemical controls after the introduction of the drug are recommended after 3 months of therapy. At our center, we used to routinely hospitalize patients for the introduction of ETI in treatment. This gave us the opportunity to monitor the onset of any early adverse events and to evaluate early changes in hematological parameters. Further studies on a larger number of patients are needed to better understand the possible mechanisms involved in Hb changes in patients taking CFTR modulators.

## Figures and Tables

**Table 1:** Comparison of laboratory parameters in 35 children with CF at the baseline and after LI therapy versus age/sex-matched healthy controls: short-term (A) and long-term (B) effects.

A	HCs	CF			
		Baseline	3 days of LI	1 month of LI	3 months of LI
Hemoglobin (g/dL)	13.7 (13.1–14.0)	13.0 (12.0–14.1)	13.9 (13.4–14.5) ^a,b^	13.1 (12.8–13.8) ^c^	13.7 (12.7–14.2) ^b^
B	HCs	CF			
		Baseline	6 months of LI	9 months of LI	1 year of LI
Hemoglobin (g/dL)	13.7 (13.1–14.0)	13.0 (12.0–14.1)	13.5 (12.9–14.5) ^b^	13.6 (13.1–13.9)	13.4 (12.6–14.1)

Data are reported as median (interquartile range). ^a^  *p* < 0.01 vs. HCs, Mann–Whitney U test; ^b^  *p* < 0.01 vs. baseline, ^c^  *p* < 0.01 vs. 3 days, Friedman test. HCs: healthy controls.

**Table 2:** Comparison of laboratory parameters in 60 children with CF at baseline and after ETI therapy versus age/sex-matched healthy controls: short-term (A) and long-term (B) effects.

A	HCs	CF			
		Baseline	3 days of ETI	1 month of ETI	3 months of ETI
Hemoglobin (g/dL)	13.3 (13.0–13.9)	13.2 (12.4–13.8)	12.8 (12.0–13.6) ^a,b^	13.1 (12.5–13.5) ^b^	13.1 (12.1–13.9)
Potassium (mEq/L)	4.4 (4.2–4.6)	4.4 (4.2–4.8)	4.6 (4.4–4.8) ^a^	4.4 (4.3–4.4) ^c^	4.3 (4.1–4.5) ^c^
Total bilirubin (mg/dL)	0.30 (0.22–0.41)	0.30 (0.22–0.42)	0.36 (0.28–0.49) ^a^	0.49 (0.38–0.66) ^a,b,c^	0.61 (0.41–0.80) ^a,b,c^
Conjugated bilirubin (mg/dL)	0.12 (0.10–0.19)	0.15 (0.12–0.18)	0.17 (0.13–0.22) ^a^	0.23 (0.17–0.29) ^a,b,c^	0.27 (0.20–0.36) ^a,b,c^
B	HCs	CF			
		Baseline	6 months of ETI	9 months of ETI	1 year of ETI
Hemoglobin (g/dL)	13.3 (13.0–13.9)	13.2 (12.4–13.8)	13.4 (12.5–13.8)	13.2 (12.7–13.8)	13.5 (12.7–14.1)
Potassium (mEq/L)	4.4 (4.2–4.6)	4.4 (4.2–4.8)	4.3 (4.1–4.5) ^b^	4.3 (4.1–4.5) ^b^	4.4 (4.2–4.6)
Total bilirubin (mg/dL)	0.30 (0.22–0.41)	0.30 (0.22–0.42)	0.62 (0.42–0.88) ^a,b^	0.60 (0.39–0.82) ^a,b^	0.49 (0.34–0.81) ^a,b^
Conjugated bilirubin (mg/dL)	0.12 (0.10–0.19)	0.15 (0.12–0.18)	0.28 (0.19–0.37) ^a,b^	0.26 (0.18–0.31) ^a,b^	0.23 (0.16–0.35) ^a,b^

Data are reported as median (interquartile range). ^a^  *p* < 0.01 vs. HCs, Mann–Whitney U test; ^b^  *p* < 0.01 vs. baseline, ^c^  *p* < 0.01 vs. 3 days, Friedman test. HCs: healthy controls.

## Data Availability

The original contributions presented in the study are included in the article, further inquiries can be directed to the corresponding authors.

## Abbreviations

CF	cystic fibrosis
CFTR	cystic fibrosis transmembrane conductance regulator
LI	lumacaftor/ivacaftor
ETI	elexacaftor/tezacaftor/ivacaftor
Hb	hemoglobin
Ht	hematocrit
K	potassium
TB	total bilirubin
CB	conjugated bilirubin
